# Supplementary material for: A Sacrificial 3D Printed Vessel‐on‐Chip Demonstrates a Versatile Approach to Model Granulation Tissue
Source: Adv Healthc Mater. 2025 Nov 21;15(7):e03081. doi: 10.1002/adhm.202503081 (PMC12908207; doi:10.1002/adhm.202503081)
Supplement: Supplementary file 1 — Supporting File 1: adhm70506‐sup‐0001‐SuppMat.docx. [file ADHM-15-0-s001.docx]

Supporting Information

**A Sacrificial 3D Printed Vessel-on-Chip Demonstrates a Versatile Approach to Model Granulation Tissue**

*Jonas Jäger, Phil Berger, Andrew I. Morrison, Hendrik Erfurth, Maria Thon, Eva-Maria Dehne, Susan Gibbs and Jasper J. Koning**

*correspondence: jj.koning@amsterdamumc.nl

**
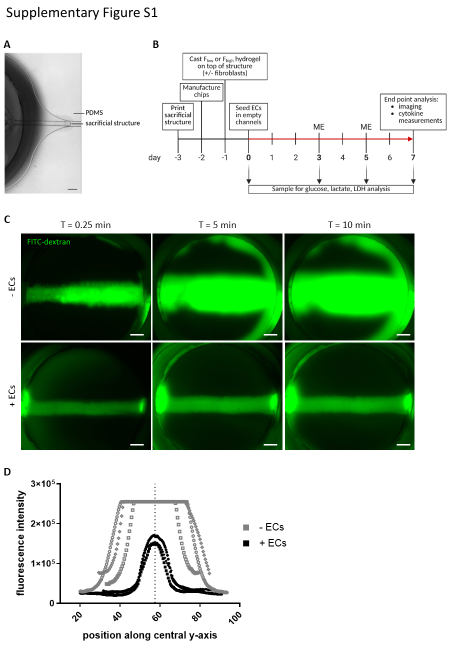
**

**Figure S1: PVA structure fit, experimental timeline, and barrier properties of the vessel-on-chip.** Additional information for Figure 1. **(A)** Bright-field image of the PVA-PDMS interface shows fit of the sacrificial structure in the PDMS channel. Scale bar: 1000 µm. **(B)** Experimental timeline for the generation of the vessel-on-chip. Chips were produced with a printed sacrificial structure incorporated after which the hydrogel (+/- fibroblasts) and ECs were added. Subsequently, the vessels were perfused for 7 days. **(C)**. FITC-dextran perfusion through the channel with or without ECs after 0.25, 5 and 10 min. Scale bars: 800 µm. **(D)** Quantification of FITC-Dextran fluorescent intensity along the central y-axis in images of three different channels in three different chips (indicated with different symbols) with or without ECs after 10 minutes of perfusion. Dashed line indicates the center of the channel. ECs: endothelial cells, ME: medium exchange, LDH: lactate dehydrogenase, F_high_: high fibrinogen, F_low_: low fibrinogen hydrogel concentrations.


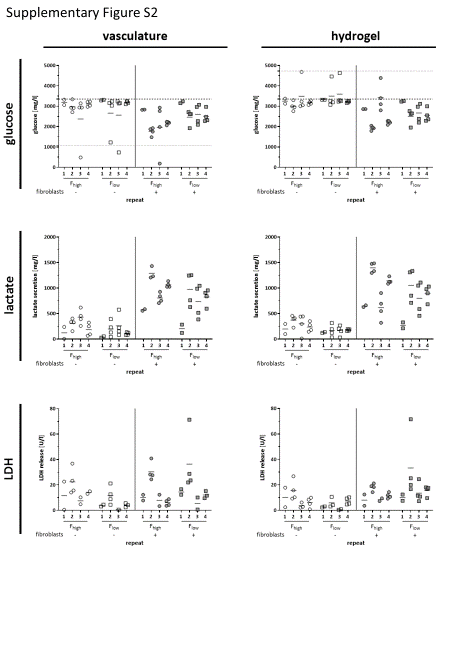


**Figure S2: Metabolic markers of EC-fibroblast co-cultures show low variability between different repeats.** Individual repeats of Figure 6. Variability between different repeats and inter-experimental variability of a total of n = 72 (≙ 36 chips) individual measurements at day 7. For vasculature and hydrogel measurements of glucose, lactate and LDH, four conditions are shown with intra-experimental replicates (each column) and repeats (1-4). Dotted lines indicate basal glucose levels of respective vasculature and hydrogel medium and dashed lines the basal glucose levels in the whole chip. Symbols represent different conditions and line mean of intra-experimental replicates.


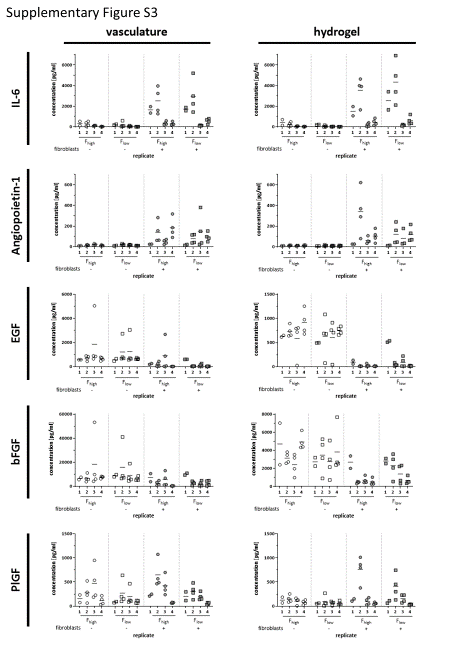


**
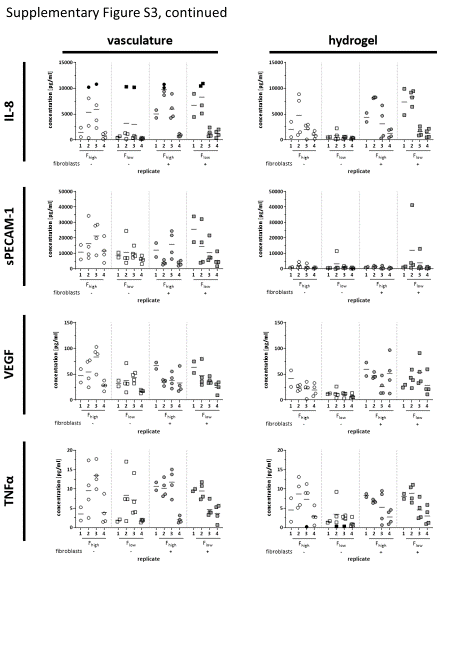
**

**Figure S3: Variability of cytokine secretion.** Individual repeats of Figure 5. Measurement of 9 different cytokines shows variability between different repeats and inter-experimental variability of a total of N = 72 (≙ 36 chips). Shown are individual measurements at day 7. Black data points were outside the detection limit, extrapolated in this figure and set to detection limit in Figure 5.

**
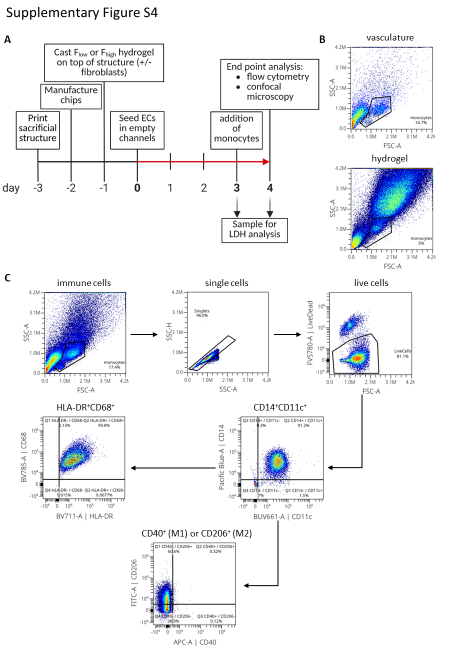
**

**
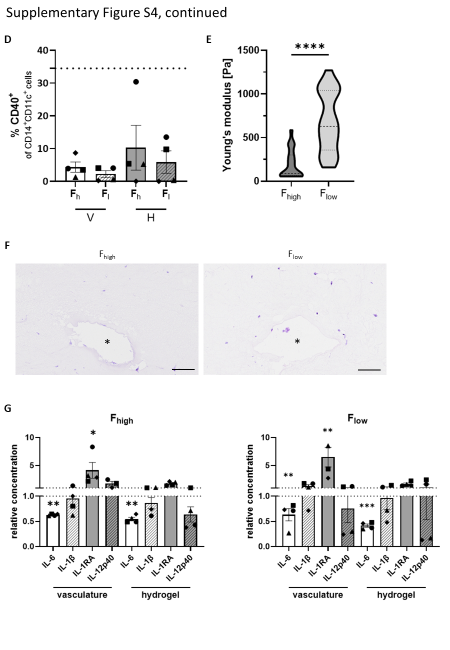
**

**Figure S4: Experimental timeline, gating strategy, histological sections and stiffness measurements for monocytes and macrophages circulated through the vessel-on-chip.** Addition to Figure 7. **(A)** Experimental timeline for 24 h monocyte perfusion on-chip. **(B)** Monocytes were directly gated based on size and granularity (FSC-SSC) to initially remove other cell types such as fibroblasts and ECs together with debris. Hydrogel and vasculature sample of the same circulation is shown as an example. **(C)** Gating was set on cells, single cells, live cells, CD14^+^CD11c^+^ cells, HLADR^+^CD68^+^ and CD40^+^ or CD206^+^ for M1 and M2 macrophages. FSC: forwards scatter, SSC: side scatter. **(D)** Percentage of CD40^+^ cells within the CD14^+^CD11c^+^ cell population. Dotted line represents marker expression of a static control. F_h_: high fibrinogen, F_l_: low fibrinogen hydrogel concentration, V: vasculature, H: hydrogel. Symbols represent different donors and columns represent mean ± SEM; n = 4 independent experiments. **(E)** Stiffness of different hydrogel conditions, measured after 4 days in the chip with fibroblasts and ECs and 24 hours of monocyte perfusion. Each condition with n ≥ 20 data points. Unpaired t-test, ****p < 0.0001. **(F)** Cross-sectional H&E staining of F_high_ and F_low_ hydrogels with fibroblasts, ECs, and monocytes. The perfusable endothelialized channel is shown in the center (*). Images were taken at day 4 after 24-hour perfusion of monocytes. H&E: hematoxylin & eosin. Scale bars: 100 µm. **(G)** Relative cytokine secretion of IL-6, IL-1β, IL‑1RA and IL‑12p40 in the vasculature and hydrogel compartment of F_high_ and F_low_ hydrogels expressed as the ratio measured before monocyte addition (day 3) and 24 hours after monocyte addition (day 4). Symbols represent different donors and columns represent mean ± SEM, measured in duplicates.


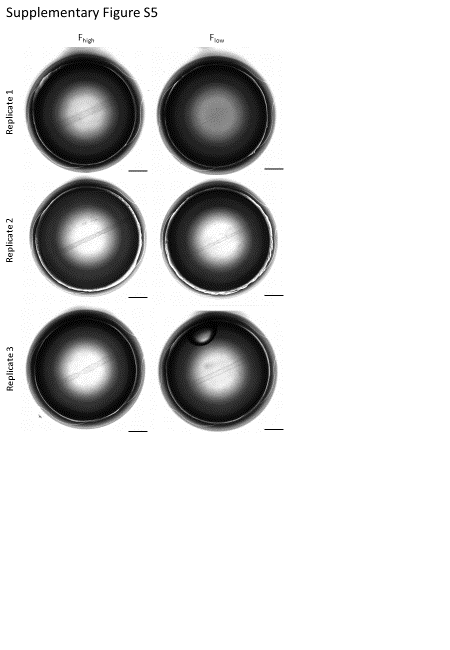


**Figure S5: Hydrogel characterization and quality-control.** Gross bright-field images of F_high_ and F_low_ hydrogels in chip with ECs and fibroblasts at day 7. Shown are representative images for 3 different replicates. Scale bars: 2000 µm.

**
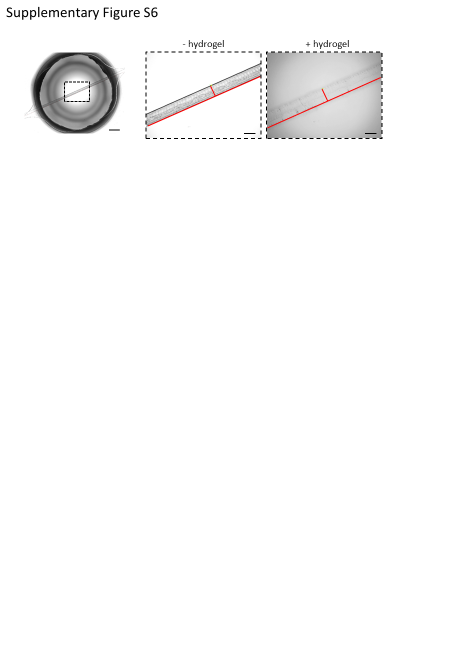
**

**Figure S6: Vessel width measurements.** Additional information for Figure 4C. Addition of a hydrogel on top of the sacrificial structure induced widening of the channel. Images were taken before addition of the hydrogel and after 24 hours. Red lines indicate channel alignment and perpendicular width measurement. Scale bars: 1000 µm in compartment overview on the left, 500 µm in inlays on the right.

**Movie S1: Hydrogel channel rendering.** ECs were stained with vimentin (yellow) and nuclei with DAPI (blue) at day 7. Surface-rendering is shown in red.
